# Neuroanatomical correlates of biological motion detection

**DOI:** 10.1016/j.neuropsychologia.2012.11.027

**Published:** 2013-02

**Authors:** Sharon Gilaie-Dotan, Ryota Kanai, Bahador Bahrami, Geraint Rees, Ayse P. Saygin

**Affiliations:** a Institute of Cognitive Neuroscience, University College London, London, UK; b Wellcome Trust Centre for Neuroimaging, University College London, London, UK; c School of Psychology, Sackler Centre for Consciousness Science, University of Sussex, Falmer, UK; d Department of Cognitive Science and Neurosciences Program, University of California San Diego, La Jolla, CA, USA

**Keywords:** Voxel based morphometry, Individual differences, Point light displays, Temporal cortex, Premotor cortex

## Abstract

Biological motion detection is both commonplace and important, but there is great inter-individual variability in this ability, the neural basis of which is currently unknown. Here we examined whether the behavioral variability in biological motion detection is reflected in brain anatomy. Perceptual thresholds for detection of biological motion and control conditions (non-biological object motion detection and motion coherence) were determined in a group of healthy human adults (*n*=31) together with structural magnetic resonance images of the brain. Voxel based morphometry analyzes revealed that gray matter volumes of left posterior superior temporal sulcus (pSTS) and left ventral premotor cortex (vPMC) significantly predicted individual differences in biological motion detection, but showed no significant relationship with performance on the control tasks. Our study reveals a neural basis associated with the inter-individual variability in biological motion detection, reliably linking the neuroanatomical structure of left pSTS and vPMC with biological motion detection performance.

## Highlights

► We examined neuroanatomical correlates of biological motion detection ability. ► Left pSTS and vPMC's neural structure predicted biological motion detection ability. ► These region's neural structure did not predict performance on the control tasks.

## 1 Introduction

Perceiving and understanding biological motion – the movements of animate entities – is important for many tasks of biological significance, from hunting prey and avoiding predators, to imitation, social cognition, and theory of mind (Blake & Shiffrar, 2007). Key to these is detecting biological motion in the environment. There is great inter-individual variability in biological motion detection ability (Chandrasekaran, Turner, Bulthoff, & Thornton, 2010; Gilaie-Dotan, Bentin, Harel, Rees, & Saygin, 2011; Hiris, 2007; Saygin, Cook, & Blakemore, 2010; van Kemenade, Muggleton, Walsh, & Saygin, 2012), yet the perceptual and neural correlates of this inter-individual variability are largely unknown.

Here we investigated the neural correlates of this inter-individual variability in biological motion detection by examining whether the neural structure of brain areas that support biological motion processing was associated with biological motion detection ability. We used an individual differences approach, a robust and reliable method with which to study associations between behavior and underlying neuronal mechanisms (Kanai & Rees, 2011; Vogel & Awh, 2008). Perceptual thresholds for biological motion detection, as well as for two non-biological motion control tasks were measured, using adaptive psychophysical paradigms in a group of healthy human adults (*n*=31). Using structural magnetic resonance imaging (MRI) and voxel-based morphometry (VBM), we then examined the relationship between behavioral performance in biological motion detection and the gray matter volume of brain regions associated with biological motion processing (Grosbras, Beaton, & Eickhoff, 2012). We also examined whether such relationships exist between gray matter volume and the control tasks.

A network of brain areas supports biological motion processing (e.g., (Grosbras et al., 2012; Grossman et al., 2000; Pelphrey, Morris, Michelich, Allison, & McCarthy, 2005; Peuskens, Vanrie, Verfaillie, & Orban, 2005; Saygin, Wilson, Hagler, Bates, & Sereno, 2004)). The posterior superior temporal sulcus (pSTS) is thought to play a key role in processing biological motion (Oram & Perrett, 1996; Puce & Perrett, 2003; Wyk, Hudac, Carter, Sobel, & Pelphrey, 2009). Ventral premotor cortex (vPMC) has also been functionally linked to biological motion processing (De Lussanet et al., 2008; Furl et al., 2010; Michels, Kleiser, de Lussanet, Seitz, and Lappe, 2009; Saygin, 2007), possibly due to multisensory (mirror) neurons in this region that are involved in action understanding (Rizzolatti & Craighero, 2004). Based on these studies and on prior neuropsychological and brain stimulation studies (De Lussanet et al., 2008; Furl et al., 2010; Michels et al., 2009; Saygin, 2007), we hypothesized that the neuroanatomical structure of the pSTS and vPMC would predict individual differences in biological motion detection.

## 2 Methods

### 2.1 Participants

31 healthy adults participated in the studies (mean age 23.7±3.93 (S.D.), 17 females). All participants had normal or corrected to normal vision, were right handed, and had no history of neurological disorders. Psychophysical experiments were conducted at the Institute for Cognitive Neuroscience, University College London; MRI scanning took place at the Wellcome Trust Centre for Neuroimaging, University College London. Participants gave written informed consent to take part in the studies, and the experiments were approved by the local ethics committee.

We conducted three behavioral experiments. In Experiment 1, we assessed biological motion detection (Bio-Det). Experiments 2 and 3 were control studies, where we estimated non-biological object motion detection (NonBio-Det) and motion coherence (MotionCoh) thresholds.

### 2.2 Experiment 1: Biological motion detection (Bio-Det)

#### 2.2.1 Stimuli

**Fig. 1:**
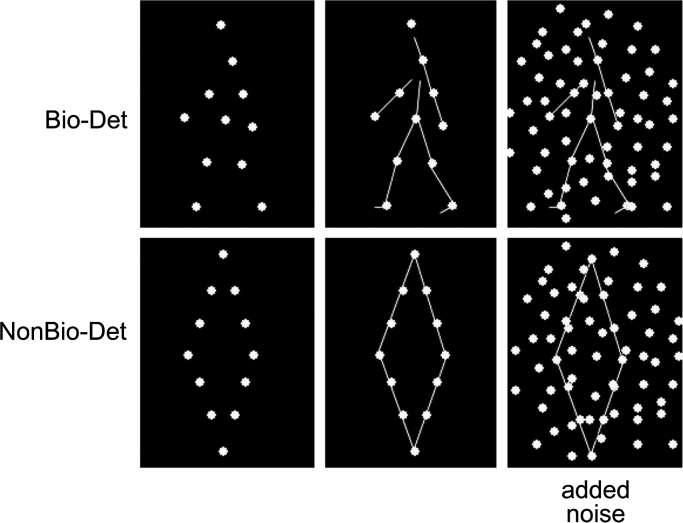
Stimuli and noise masking of Experiments 1 and 2. Still frames from the animations are shown without any noise (left) and with one level of noise points (right). Top: an example of a biologically moving figure (walker) from Experiment 1 (Bio-Det), bottom: an example of a non-biologically moving object (diamond) from Experiment 2 (NonBio-Det). The connecting stick lines are added as a visual aid here and were not present in the experiments. The moving noise points were added to the stimuli in Experiments 1 and 2 in an adaptive manner to determine individual perceptual thresholds (i.e., the estimated number of noise points for 75% accuracy) using a Bayesian adaptive method, QUEST (Watson & Pelli, 1983). The more noise points were added, the more difficult the task became. Noise points in the biological motion animations (top) had the same motion trajectories as the target point-light animation. In the non-biological animations (bottom) the noise points translated at the same speed as the target object, half in the opposite direction (see Section 2). In the experiments participants had to decide in each trial if a target stimulus was present or absent (see Section 2). Present and absent stimuli had same local motion. See Section 2 for further details.

In the laboratory, biological motion processing is typically assessed with the well-established point-light biological motion stimuli (similar to those reported by (Johansson, 1973)), which track movements of a small number of joints on the body with points (Fig. 1). Despite the sparse form information they provide, when in motion, these stimuli elicit a clear depiction of body movements and actions, even conveying gender, identity, and emotional state of the actor (Blake & Shiffrar, 2007).

The biological motion stimuli used here along with the adaptive noise-masking paradigm (see below) were successfully used in previous biological motion detection studies (Gilaie-Dotan et al., 2011; Grossman et al., 2000; Kim, Park, & Blake, 2011; Saygin et al., 2010). These stimuli were created by videotaping an actor performing various activities, and encoding the joint positions with point-lights in digitized videos (Ahlstrom, Blake, & Ahlstrom, 1997). Animations depicted 12 human motions (walking, jogging, stepping up (climbing stairs), lifting knee across, low kicking (football), kicking across, high kicking sideways, three pitching throws, underarm throwing (bowling), and rope skipping). The joints of the actor were represented by 12 small white points against a black background (Fig. 1) and could be briefly invisible due to occlusion by other body parts.

Each of the 12 biological motion animations had a matched “scrambled” animation that contained the same local motion information, but without the global form (e.g., Bertenthal & Pinto, 1994; Gilaie-Dotan et al., 2011; Grossman et al., 2000; Kim et al., 2011; Saygin et al., 2010, 2004). This was achieved by spatially scrambling the starting positions of the 12 points of each animation while keeping their motion trajectories intact. For each animation, the starting positions were chosen within a region such that the area occupied by the scrambled point-light display was similar to that for the original biological motion point-light display. The scrambled point-light animations were used for the target-absent trials in the biological motion detection task (see below). Each animation subtended approximately 5.5×7.7 deg visual angle when viewed from 52 cm. In each trial, biological motion animations were presented along with additional noise points (see adaptive thresholding below), altogether subtending approximately 8×12 deg visual angle.

Stimuli were presented on a 60 Hz screen with 1024×768 resolution, and responses recorded using MATLAB (Mathworks, Inc) and the Psychophysics Toolbox V2.54 (Brainard, 1997; Pelli, 1997).

#### 2.2.2 Noise masking and adaptive thresholding

Since we were interested in individual differences, we used adaptive methods to estimate thresholds that were dependent measures in our analyzes. Advantages of adaptive thresholding include efficiency, relative protection from ceiling and floor effects, and applicability to different age groups or clinical populations.

To obtain a psychometric measure of performance, we used established methods to add noise (i.e., extra moving points) to point-light displays (Fig. 1) (Bertenthal & Pinto, 1994; Gilaie-Dotan et al., 2011; Grossman et al., 2000; Hiris, 2007; Ikeda, Blake, & Watanabe, 2005; Kim et al., 2011; Saygin, 2007; Saygin et al., 2010). The more noise points were added, the more difficult it became to perform these perceptual tasks. The noise points moved with the same trajectories as points from the target animations. In each trial, the target point-light animation was presented along with an adaptively varied number of noise points determined through a Bayesian estimation based on the participant's responses until that trial. We estimated the number of noise points at which each participant performed at 75% accuracy using the QUEST algorithm (Watson & Pelli, 1983). QUEST initial priors were an initial threshold guess based on log_10_(20 dots) with a standard deviation of 0.4, threshold criterion of 0.75, Weibull psychometric function parameters beta=3.5, delta=0.02, and gamma=0.5.

#### 2.2.3 Experimental procedures and task

At the beginning of the experiment, participants completed a 12 to 20 trial practice session, which featured a range of predetermined number of noise points (ranging from 0 to 70). All participants understood the task after the practice. After the practice, each participant completed two blocks of 68 trials with the adaptive QUEST procedure beginning with 20 noise points, with a target accuracy of 75%. A 10-s break followed trial 36 in each block, and additional rest was allowed between blocks. Each block lasted 3–4 min.

Each trial started with a fixation cross appearing for 750 ms, after which the point light stimulus (the target stimulus or its scrambled counterpart, plus the noise points) appeared for 667 ms (40 frames). After the participant's response was recorded, feedback was provided via the color of the fixation cross (green for correct, red for incorrect) that appeared for 750 ms. If no response was given within 2000 ms from the end of stimulus presentation, an incorrect response was used for the adaptive algorithm. The next trial began with the number of noise points estimated by QUEST. On each trial, the target point-light display (or its scrambled counterpart) was presented at a randomly jittered location within a 2.2 deg radius from the centre of the screen in order to prevent a response strategy based on purely local information.

The participants' task was to detect whether or not the trial contained the target object (a biological motion animation of an upright human figure performing an action) and to respond by pressing one of two keys with their right hand.

### 2.3 Experiment 2: Non-biological object motion detection (NonBio-Det)

#### 2.3.1 Stimuli

We examined whether any correlations between biological motion detection thresholds and neuroanatomy generalized to individual differences in the detection of non-biologically moving objects. To this end, we kept the participants' task and all other methodological details matched with Experiment 1, but instead of biological motion, we now used non-biologically moving point-light stimuli depicting geometric shapes (Fig. 1, (De-Wit, Lefevre, Kentridge, Rees, & Saygin, 2011; Hiris, 2007; Saygin et al., 2010; van Kemenade et al., 2012)).

Twelve point-lights of the same size, shape and color as those in the biological motion animations were used to define four-sided polygons (square, rectangles, diamonds, rhombus and parallelograms). These stimuli moved non-biologically, translating at a fixed speed of 0.5 pixels/frame, corresponding approximately to the speed of the points of the biological motion stimuli. For each of the 12 non-biological point-light shapes, we also generated a “scrambled” equivalent to be presented in the target-absent trials. In the scrambled stimuli, the same number of points translated with the same motion trajectories as the target shapes, but with the positions of the points scrambled such that they did not comprise a recognizable shape. The presentation setup including visual angles of the stimuli was closely matched to Experiment 1.

#### 2.3.2 Noise masking and adaptive thresholding

We used the same methods as Experiment 1 to estimate perceptual thresholds for non-biological object motion detection. A variable number of noise points that translated in either direction were added to each trial and noise point thresholds were estimated adaptively (Gilaie-Dotan et al., 2011; Hiris, 2007; Kim et al., 2011; Saygin et al., 2010; Watson & Pelli, 1983).

#### 2.3.3 Experimental procedures and task

Experimental details including presentation timings were precisely matched to Experiment 1. Each participant was required to detect whether or not the trial contained the target object (a translating point-light polygon) that appeared for 667 ms (see more details in Section 2.2.3), and then respond by pressing one of two keys on the keyboard with their right hand.

### 2.4 Experiment 3: Motion coherence (MotionCoh)

#### 2.4.1 Stimuli

Non-object-based non-biological motion processing was assessed using motion coherence thresholds. Stimuli were circular random dot patterns (Green, 1961; Levinson & Sekuler, 1976) displayed in the center of the screen. Each stimulus consisted of 500 grey points (luminance=2.50 Cd/m^2^, width=2.77 minArc) against a black background (luminance=0.37 Cd/m^2^) covering a circular area (width=9.13 deg when viewed from 52 cm). A two-interval forced choice (2IFC) paradigm was employed. In each trial, two stimuli intervals were presented (each interval for 333 ms with inter stimulus interval=1000 ms). One randomly chosen interval consisted of coherent motion plus noise and the other interval only consisted of noise. For the signal plus noise stimulus, a randomly chosen subset of the dots was vertically displaced upwards or rightwards by 0.45 deg steps in twenty consecutive frames (total motion time=333 ms; speed=27.27 deg/s). The rest of the points were repositioned randomly from one frame to the next. Coherently moving points reaching either end of the display area were repositioned on the other side for the next frame. A central fixation square (width=0.55 deg) was displayed throughout the experiment.

Stimuli were generated using the Cogent toolbox (http://www.vislab.ucl.ac.uk/cogent.php) for MATLAB (Mathworks, Inc) and were presented at 60 Hz using a TFT-LCD display (800×600 resolution).

#### 2.4.2 Adaptive thresholding

For each participant we determined the threshold coherence level that enabled 75% correct identification of target interval that contained upward motion according to an accelerated stochastic approximation method (Kesten, 1958; Treutwein, 1995). This method updates the approximation on every trial in a staircase manner, with stairs becoming smaller following a change in the response accuracy; incorrect responses have a bigger effect (“penalty”). Each run of the staircase consisted of 48 trials.

#### 2.4.3 Experimental procedures and task

Participants started with a short practice session with initial coherence level of 50% and were asked to press one of two buttons to indicate whether the first or second interval contained more coherent motion. To verify that participants understood the task and were able to perform it the practice was accompanied by verbal explanation. All participants understood the task after practice. Participants then performed three runs, first run with an initial coherence threshold of 40%. For the second and third runs, the input threshold was taken as the output of the previous run.

### 2.5 Behavioral data analysis

For Experiments 1 and 2, performance thresholds were estimated for each block using QUEST (Watson & Pelli, 1983), and the average of the two blocks was taken as the dependent variable. For Experiment 3, the average of the thresholds from the second and third runs was used as a dependent variable.

### 2.6 MRI data acquisition

MR images were acquired on a 1.5-T Siemens Sonata MRI scanner (Siemens Medical, Erlangen, Germany). High-resolution anatomical images were acquired using a T1-weighted 3-D Modified Driven Equilibrium Fourier Transform (MDEFT) sequence (TR=12.24 ms; TE=3.56 ms; field of view=256×256 mm^2^; voxel size=1×1×1 mm). During scanning, head motion was restrained by padding inserted between the participant's head and the head coil.

### 2.7 Structural MRI voxel-based morphometry analyzes

For each participant the T1-weighted MR images were imported into SPM8 with NIfTI format (http://nifti.nimh.nih.gov/nifti-1). All the images were re-centred to the anterior commissure to avoid segmentation failures that can occur when the origin of the image is far from the central part of the brain. Images were then segmented into gray matter (GM), white matter (WM), and cerebrospinal fluid, using the standard segmentation procedure in SPM8 (http://www.fil.ion.ucl.ac.uk/spm) with all the recommended default parameters. The segmented gray matter images were then each manually examined using the “check registration” function in SPM8 to ensure there were no segmentation failures. To perform Diffeomorphic Anatomical Registration Through Exponentiated Lie Algebra (DARTEL) in SPM8 for inter-participant registration of the GM images (Ashburner, 2007), the spatial transformation and parameter files were imported into DARTEL using the “initial import” function with its default settings. Using the DARTEL “create template” with the default parameters, the gray matter images were then warped into a template that was created based on their own mean in an iterative manner. Subsequently, using the “normalize to MNI space” DARTEL function, the registered images were smoothed with a Gaussian kernel with the default filter size (FWHM=8 mm), and transformed to MNI stereotactic space using affine and non-linear spatial normalization implemented in SPM8 (with the recommendations for VBM analyzes options: according to many subjects, and preserving the amount of signal). We followed the default smoothing filter size in the normalization step as it is the recommendation for VBM studies, due to the good coregistration between the images (achieved in the “create template” step), and since filter size of 6 mm or smaller might lead to too many false positives. Multiple regression analysis was performed for biological motion detection (Bio-Det), while age, gender and the total grey matter volume were included as covariates of no interest in the design matrix to regress out any effects attributable to them.

**Fig. 2:**
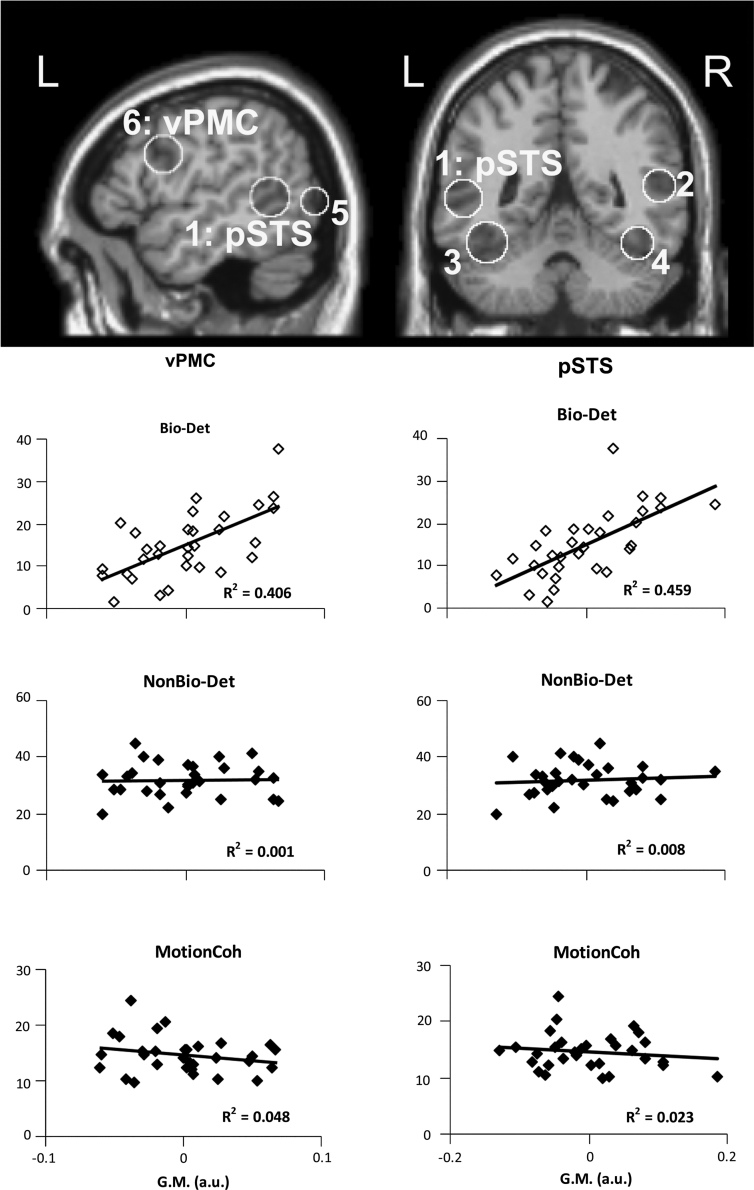
Neuroanatomical structural correlates of biological motion detection. (a) VBM analysis was performed on regions commonly associated with biological motion (Grosbras et al., 2012) as depicted: 1/2—left/right pSTS, 3/4—left/right fusiform, 5—left lateral occipital (LO/ITG), 6—vPMC (see also Table 1). *L*/*R*—left/right hemisphere, respectively. The region of interest was defined as the union of seven spheres (10 mm radius) centered at these regions' coordinates (Grosbras et al., 2012).The neural structure of left posterior superior temporal sulcus (pSTS, indicated as region 1 on sagittal section on the left, coronal on right), and left ventral premotor cortex (vPMC, indicated as region 6 on sagittal section on the left) in the inferior precentral sulcus showed significant correlation to biological motion detection ability (*P*(corr.)<0.05). No other region was significantly correlated with biological motion detection performance even at lower statistical thresholds (see Table 1 and Section 2 for anatomical and statistical details). (b) Gray matter volume of the left vPMC and pSTS foci does not correlate with performance in control tasks. Correlation between the average gray matter volume of the significant clusters (G.M., in arbitrary units on the *x*-axis) of the left vPMC (left column) and pSTS (right column) and perceptual thresholds for biological motion detection (Bio-Det, top), non biological object detection (NonBio-Det, middle), and motion coherence (MotionCoh, bottom). Perceptual thresholds (on the *y*-axis) for Bio-Det and NonBio-Det are estimated in noise points, for MotionCoh in percentage of coherent motion. Each point in the scatter plots represents data from one participant. The correlation of Bio-Det with grey matter volume (top, empty circles) is not inferential (due to circular reasoning), and is only presented for descriptive purposes. Gray matter volume was not significantly correlated with the control conditions (all *p*’s>0.23). The coefficient of determination (*R*^2^) of each correlation is presented on the bottom right of the plot.

We focused our analyzes on the loci commonly implicated in the processing of biological motion in previous work. These regions of interest were selected based on a recent meta-analysis of biological motion perception studies (Grosbras et al., 2012) and are all listed in Table 1. VBM analysis was performed for a restricted volume consisting of the union of seven spheres (10 mm radius), each of which centered at one of the regions of interest coordinates. This was done by computing a *T* contrast with *P*<0.001 uncorrected as the criterion to detect voxels with significant correlation to individual's performance-level. We then restricted the analysis to our volume of interest, i.e., regions implicated in processing biological motion (the union of the seven spheres, see above) using small volume correction, to identify regions that showed a relationship between biological motion and gray matter volume. We report here in Section 3 and in Table 1 only clusters that survived a statistical threshold of *P*(corrected)<0.05 at a cluster-level with non-stationary correction (http://fmri.wfubmc.edu/cms/NS-General (Hayasaka, Phan, Liberzon, Worsley, & Nichols, 2004), see Table 1 and Fig. 2a).

To examine whether our VBM results with biological motion detection (see Fig. 2b) were also associated with behavioral performance in the control tasks, we then extracted the average gray matter volume from each significant cluster that showed an association between biological motion detection and gray matter volume (at corrected levels of significance; see above) using the MarsBar toolbox for SPM (http://marsbar.sourceforge.net, M. Brett, J. Anton, R. Valabregue, and J. Poline. Human Brain Mapping conference, Japan, 2002). We then tested whether the extracted gray matter volumes correlated with the independent behavioral measures of the control conditions (Experiments 2 and 3). We evaluated these correlations after regressing out age, gender and total gray matter volume as we did for the main VBM multiple regression.

## 3 Results

The average perceptual thresholds for biological motion detection (Bio-Det) were 15.09±7.9 (S.D.) noise points, for non-biological motion detection (NonBio-Det) 32.0±5.9(S.D.) noise points, and for motion coherence (MotionCoh) 15%±4% (S.D.), consistent with previous work employing the same paradigms for biological and non-biological perceptual threshold estimation ((Gilaie-Dotan et al., 2011) and (Miller & Saygin, 2012)). Importantly, the inter-individual variability in biological motion detection thresholds (Bio-Det, as displayed in Fig. 2B on the *y*-axis) was large (range of noise points determining threshold: 1.7–37.7), consistent with previous results (Gilaie-Dotan, Kanai, & Rees, 2011; Saygin et al., 2010; van Kemenade et al., 2012).

Our goal was to examine whether individual differences in biological motion detection performance were correlated with the neural structure of cortical regions associated with biological motion processing. We found that the grey matter volume of two clusters, one in the left posterior superior temporal sulcus (pSTS) and one in the left inferior precentral sulcus in ventral premotor cortex (vPMC), was significantly correlated (*P*(corr.)<0.05) with biological motion detection ability (Bio-Det, main experiment), as shown in Fig. 2 (see Table 1 for full anatomical and statistical details). A further examination at a more lenient threshold in our volume of interest (*P*(uncorrected)<0.01), and at a whole-brain corrected level (*P*(corr.)<0.001), did not reveal additional regions whose neuroanatomical structure significantly correlated with biological motion detection.

To examine whether the relationship we found between gray matter density and behavioral measures was specific to biological motion detection ability, we then extracted the grey matter volume of these clusters within pSTS or vPMC that were correlated with biological motion ability, and regressed them onto independent behavioral data from Experiments 2 and 3 reflecting non-biological motion detection and motion coherence thresholds (see Fig. 2b). These behavioral data from the control conditions (NonBio-Det, MotionCoh) did not show a correlation with grey matter density of the pSTS cluster (all *t*(29)<0.84, *p*>0.41) or the vPMC cluster (all *t*(29)<1.22, *p*>0.23). Thus, the structure of both pSTS and vPMC were significantly correlated with the performance of biological motion detection, and not with those of the control tasks.

Thus, individual differences in the neuroanatomy of regions functionally linked to biological motion were predictive of individual differences in biological motion detection, but not for the detection of non-biologically moving stimuli, or motion coherence thresholds.

## 4 Discussion

The detection of biological motion is key to achieving many important and ubiquitous tasks in our daily lives. Although there are notable differences between individuals in biological motion detection ability, the neural correlates of this variability had not been explored. Here, we used voxel-based morphometry (VBM), a method that has been successfully used in relating individual differences in perception and cognition with individual differences in neuroanatomy (Fleming, Weil, Nagy, Dolan, & Rees, 2010; Gilaie-Dotan, Harel, Bentin, Kanai, & Rees, 2012; Gilaie-Dotan et al., 2011; Kanai, Bahrami, & Rees, 2010; Kanai & Rees, 2011; Maguire et al., 2000; Schwarzkopf, Song, & Rees, 2011).

We found that the neuroanatomical structure of two regions that had previously been functionally linked to biological motion processing – the posterior superior temporal sulcus (pSTS) and ventral premotor cortex (vPMC) – could reliably predict individually estimated perceptual thresholds for biological motion detection. The neuroanatomical structure of these regions did not show significant correlations with performance in two different non-biological motion control tasks.

The pSTS and vPMC are functionally linked to biological motion perception in a number of studies (Blake & Shiffrar, 2007; Saygin, in press). Electrophysiological and functional neuroimaging studies show that the pSTS responds significantly more to biological motion compared to other motion stimuli (Grossman et al., 2000; Grossman, Jardine, & Pyles, 2011; Nelissen, Vanduffel, & Orban, 2006; Peuskens et al., 2005; Puce & Perrett, 2003; Saygin et al., 2004). The vPMC is also implicated in neuroimaging studies of biological motion (De Lussanet et al., 2008; Jung et al., 2009; Michels et al., 2009; Michels, Lappe, & Vaina, 2005; Saygin et al., 2004; Vaina, Solomon, Chowdhury, Sinha, & Belliveau, 2001), possibly linked to motor simulation elicited by observed movements in premotor cortex, as proposed by work on primate mirror neurons (Rizzolatti & Craighero, 2004). Moreover, the pSTS and vPMC appear to play a critical functional role in biological motion processing as evidenced by lesion analyzes of stroke patients (Saygin, 2007) and by transcranial magnetic stimulation studies with healthy adults (Grossman, Battelli, & Pascual-Leone, 2005; van Kemenade et al., 2012).

The present study constitutes a significant step forward in understanding the neural basis of biological motion processing by using an individual differences approach implicating neuroanatomical structure. Although the pSTS and vPMC are functionally linked to biological motion processing, our work goes beyond this to show that regional variation in the macroscopic neuroanatomy of these areas predict individual differences in biological motion detection. Our findings converge with those of previous studies, even with different methods and participant populations, revealing that the same regions that are linked with impairments in biological motion perception when damaged, are also those that are significantly predictive of individual perceptual abilities in biological motion detection in the healthy brain. These findings contribute to the recent body of studies that have linked variation in the neural structure of the healthy brain with variation in cognitive or perceptual abilities in several other domains (Fleming et al., 2010; Gilaie-Dotan et al., 2012; Gilaie-Dotan et al., 2011; Kanai et al., 2010; Kanai & Rees, 2011; Maguire et al., 2000; Schwarzkopf et al., 2011).

We also tested whether any neuroanatomical correlates observed with biological motion detection, were also correlated with performance of the same participants in two control tasks. The neuroanatomical volumes of pSTS and vPMC did not predict behavioral performance in non-biological object detection or motion coherence judgments.

Previous fMRI studies have pointed to a possible right hemispheric bias in biological motion processing (Grossman et al., 2000; Herrington, Nymberg, & Schultz, 2011; Pelphrey et al., 2003, 2005). In other studies, there was no evidence of a laterality effect, and the left pSTS and vPMC have both been linked to biological motion processing (Saygin, 2007; Saygin et al., 2004). Here we observed only the left hemisphere regions predicted individual sensitivity to biological motion as measured by our tasks. It is likely that both hemispheres are involved in biological motion processing, and that any laterality effects in biological motion processing are relatively subtle and dependent on the specifics of the stimuli and task. Consistent with the present study, the neuropsychological and TMS studies that established functional role for the left pSTS and vPMC in biological motion processing, also employed detection tasks (De Lussanet et al., 2008; Furl et al., 2010; Michels et al., 2009; Saygin, 2007; van Kemenade et al., 2012). An alternative explanation is that whereas previous studies had examined common neural mechanisms across observers, our current study examined individual differences. The observed left lateralization in our study may possibly reflect lateralization of anatomical *variability* rather than a functional lateralization (cf. (Gilaie-Dotan et al., 2011)).

The primate pSTS is a complex brain area that in part subserves social functions (Hein & Knight, 2008; Kaiser, Shiffrar, & Pelphrey, 2011; Kanai, Bahrami, Roylance, & Rees, 2011; Sallet et al., 2011; von dem Hagen et al., 2011). vPMC is also thought to support social cognition via an embodied simulation of seen actions based on neural representation(s) of the observer's body (Rizzolatti & Fabbri-Destro, 2008). While future work is needed to establish a precise relationship between biological motion perception and social cognition (Pavlova, 2012), the present results firmly link individual sensitivity in the visual perception of biological motion to individual variation in the neuroanatomy of action and body movement processing networks (Rizzolatti & Craighero, 2004; Saygin, in press). The present data show that these regions are not only part of a network that functionally supports biological motion processing, but that their neuroanatomical structure can even account for the behavioral inter-individual variability observed in biological motion detection.

## Figures and Tables

**Table 1:** Voxel based morphometry results for biological motion detection based on regions associated with biological motion.

**Region of interest**				**VBM results (Bio-Det)**					
Anatomical description	*X*	*Y*	*Z*	*P* (corr.)	Cluster size	*Z* score	Peak		
							X	Y	Z
Right lateral occipital (LO/ITG)	50	−68	−2	N.S.					
Left lateral occipital (LO/ITG)	−44	−74	2	N.S.					
Right posterior superior temporal sulcus (pSTS)	54	−54	10	N.S.					

**Left posterior superior temporal sulcus (pSTS)**	−52	−50	4	**0.013**	800	3.73	−54	−49	10
							−51	−55	12
							−57	−46	7
Right fusiform gyrus	42	−54	−20	N.S.					
Left fusiform gyrus	−40	−48	−20	N.S.					

**Left ventral premotor cortex (vPMC)**	−50	8	28	**0.036**	30	3.36	−48	0	28

Regions of interest (ROIs) were selected from a recent meta-analysis of biological motion studies (Grosbras et al., 2012); human movement>non-human movement with ALE score>16.5 and *Z*-score>4). LO/ITG-lateral occipital area/inferior temporal gyrus. vPMC: ventral premotor cortex (in the inferior precentral sulcus), pSTS—posterior superior temporal sulcus. VBM analyzes in the volume of these regions were performed for biological motion (Bio-Det, see Section 2 and Fig. 2). *P* values are non-stationary-corrected (Hayasaka et al., 2004). Local peak coordinates are provided (multiple coordinates for peaks more than 4 mm apart within the same cluster). N.S. indicates that no supra-threshold (*P*(corr.)<0.05) clusters were found within 10 mm of these coordinates (this was also true at a more lenient threshold of uncorrected *P*<0.01). Regions showing significant effects (left pSTS and vPMC) are highlighted in bold. Cluster size indicated in mm^3^. Coordinates are in MNI space. See also Fig. 2.
